# The New Sydenham Society of London

**Published:** 1861-07

**Authors:** 


THE NEW SYDENHAM SOCIETY OF LONDON.—The Honorary Local Secretary for Philadelphia, Dr. Richard J. Dunglison, 121 South Tenth Street, has just received the volumes for members for 1860, including "Clinical Memoirs on Abdominal Tumors and Intumescence," by the late Richard Bright, M.D., F.R.S.; "A Clinical Treatise on Diseases of the Liver," vol. i., by P. T. Frerichs, M.D., Professor of Clinical Medicine in the University of Berlin; and "A Year-Book of Medicine, Surgery, and their Allied Sciences," for 1859; together with a specimen plate of the beautiful Atlas of Skin Diseases, copied from the splendid work of Hebra, published by this society; which may be seen at his office. Each member for 1860 receives the three handsome volumes first named, and the first fasciculus of the Atlas, including three life-size plates.

The annual subscription is five dollars and a quarter, payable in advance. The books are received and distributed by the secretary, immediately upon their arrival from England. The volumes for 1859 may still be obtained. The number of members has already reached several thousand. The advantages offered to each individual member by a publishing society such as this increase *pari passu* with its number of members, *i.e.* each subscriber will receive more books if the society maintains its present great success or advances beyond it, than he would under less favorable circumstances.

Considering the character and number of the works already brought out, the society certainly commends itself to the very extended support of the profession.

